# Automatic granular and spinous epidermal cell identification and analysis on *in vivo* reflectance confocal microscopy images using cell morphological features

**DOI:** 10.1117/1.JBO.28.4.046003

**Published:** 2023-04-08

**Authors:** Imane Lboukili, Georgios Stamatas, Xavier Descombes

**Affiliations:** a Johnson & Johnson Santé Beauté France, Paris, France; b UCA—INRIA—I3S/CNRS, Sophia Antipolis, France

**Keywords:** epidermis, image segmentation, reflectance confocal microscopy, object detection

## Abstract

**Significance:**

Reflectance confocal microscopy (RCM) allows for real-time *in vivo* visualization of the skin at the cellular level. The study of RCM images provides information on the structural properties of the epidermis. These may change in each layer of the epidermis, depending on the subject’s age and the presence of certain dermatological conditions. Studying RCM images requires manual identification of cells to derive these properties, which is time consuming and subject to human error, highlighting the need for an automated cell identification method.

**Aim:**

We aim to design an automated pipeline for the analysis of the structure of the epidermis from RCM images of the *Stratum granulosum* and *Stratum spinosum*.

**Approach:**

We identified the region of interest containing the epidermal cells and the individual cells in the segmented tissue area using tubeness filters to highlight membranes. We used prior biological knowledge on cell size to process the resulting detected cells, removing cells that were too small and reapplying the used filters locally on detected regions that were too big to be considered a single cell. The proposed full image analysis pipeline (FIAP) was compared with machine learning-based approaches (cell cutter, different U-Net configurations, and loss functions).

**Results:**

All methods were evaluated both on simulated data (four images) and on manually annotated RCM data (seven images). Accuracy was measured using recall and precision metrics. Both accuracy metrics were higher in the proposed FIAP for both real (precision=0.720±0.068, recall=0.850±0.11) and synthetic images (precision=0.835±0.067, recall=0.925±0.012). The tested machine learning methods failed to identify and segment keratinocytes on RCM images with a satisfactory accuracy.

**Conclusions:**

We showed that automatic cell segmentation can be achieved using a pipeline based on membrane detection, with an accuracy that matches expert manual cell identification. To our knowledge, this is the first method based on membrane detection to study healthy skin using RCM images evaluated against manually identified cell positions.

## 1 Introduction

Reflectance confocal microscopy^1^^,^^2^ (RCM) is a real-time noninvasive *in vivo* technology that allows for the visualization of the skin epidermis and upper layers of the dermis at the cellular level. It is noninvasive, thus making it a technique of choice for repeated sampling on a skin site without damage, when studying the changes in skin structure over time or when an invasive biopsy cannot be considered, e.g., in the study of healthy baby skin physiology. Images are formed by scanning a laser light source in a plane parallel to the skin surface and collecting the back-scattered light. Light scattering events occur at the interface of microstructures with different indices of refraction. In skin, such microstructures are keratin fibers, melanosomes, collagen fibers, and cell membranes. Therefore, it provides information on the geometrical (e.g., projected cell area and cell perimeter) and topological (e.g., cell density and number of nearest neighbors) properties of the skin, which play important roles in the architecture of the skin barrier.

In most cases, analysis of RCM stacks is done manually, providing qualitative observations. However, manual analysis is time consuming, intensive, and subject to human interpretation and interexpert differences. Thus, we could benefit from automated methods to quantitatively analyze RCM images. An important first step in any quantitative study of skin is cell detection. Unfortunately, it is challenging and requires a robust generic algorithm to alleviate nonuniformity and noise inherent to RCM images.

The epidermis is made of four distinct layers. From the deepest to the most superficial, they are *Stratum basale, Stratum spinosum, Stratum granulosum*, and *Stratum corneum.*

In RCM images of light-pigmented skin, the *S. corneum* appears as large bright islands surrounded by dark areas representing the skin microrelief lines. It is made of dead anucleated but biochemically active cells.^3^ As we cannot observe individual cells on RCM images of the *S. corneum*, our method will not be applied to these images.

The *S. granulosum* and *S. spinosum* appear as agglomerations of viable keratinocytes arranged in a honeycomb pattern.^4^ Granular cells are typically larger than spinous cells, and as such they have a lower density.^5^

Finally, the *S. basale* is made of the smallest keratinocytes; as their differentiation starts in the *S. basale* and continues as cells migrate toward the skin surface, their enface cross-section area gets larger as the cells become flatter. The *S. basale* is attached to the dermis on the dermal–epidermal junction, and thus we can sometimes observe the top of dermal papillae on RCM images of the *S. basale*. In addition, melanin-producing melanocytes are scattered through the basal layer. Organelles filled with melanin, called melanosomes, are transferred from melanocytes to keratinocytes. Illumination light intensity drops almost exponentially as a function of depth in the tissue because of light losses in back-scattering events. Due to this phenomenon and because the basal layer is the deepest epidermal layer, images in the *S. basale* display more noise and appear of lower quality than images of the *S. granulosum* and *S. spinosum*. For these reasons, we focus on the granular and spinous layers in our attempt at automating the detection of keratinocytes.

**Fig. 1:**
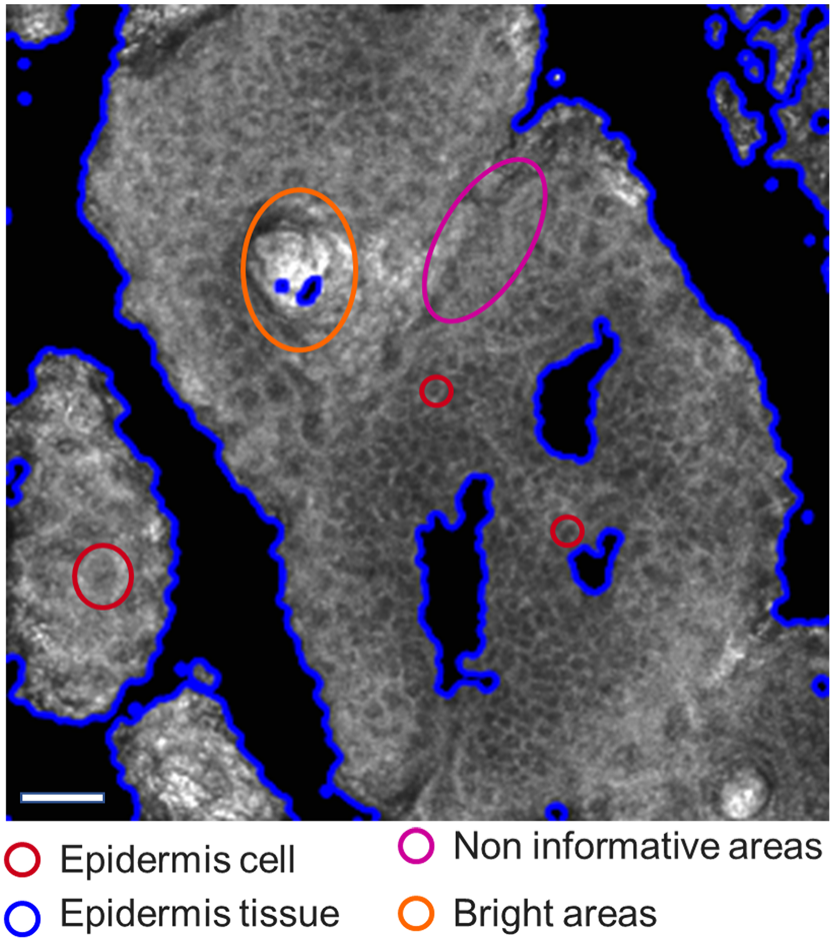
RCM image of the *S. spinosum* of minimally pigmented skin, Fitzpatrick type II. In blue, the border between tissue and background formed by micro-relief lines. Area marked in pink, noninformative areas; in orange, bright spots; in red, epidermal cells. Image contrast was adjusted for better visualization. Scale bar = 100 microns.

On RCM images of the *S. granulosum* and *S. spinosum* of minimally pigmented skin, keratinocytes are characterized by a dark center and a grainy cytoplasm due to microstructures and are surrounded by bright grainy membranes (see Fig. 1).

Previously, few attempts at the automated identification of epidermal cells on RCM images have been made.^6^^,^^7^ Unfortunately, the amount of noise and heterogeneity of RCM images hinders the development of accurate segmentation methods. Here, we propose a method to automatically detect keratinocyte positions on RCM images of the *S. granulosum* and *S. spinosum*. We compare our results to a ground truth of cell center positions obtained manually and achieve an accuracy on par with expert graders.

## 2 Data

*In vivo* RCM images were captured on the volar forearm of 80 participants: 60 children aged 3 months to 10 years old and 20 adults aged 25 to 40 years old. All participants have minimally pigmented skin, with Fitzpatrick types between I and III (2.5%, 87.5%, and 10% of participants had Fitzpatrick type I, II, and III, respectively). Inclusion criteria required that the participants were in good health, with no history of skin disease, and had not applied any products on the observed area the day of the study. Only 7 images (4 participants, 20 to 35 years) were used in the algorithm development and validation.

Images were captured using a Vivascope 1500 reflectance confocal microscope with a z-resolution of 5  μm and xy-resolution of 1  μm. Images started at the *S. corneum* and progressed down toward the *S. basale*. The size of each image is 1000×1000  pixels, corresponding to $500\times 500\;\mu m^{2}$.

Each image was classified in one of the four epidermal layers using a hybrid deep learning algorithm^8^ trained on 1500 images to classify RCM images into six categories, i.e., outside of skin, *S. corneum*, *S. granulosum*, *S. spinosum*, *S. basale*, and dermis with a test accuracy of 82%, allowing us to focus only on images of the *S. granulosum* and *S. spinosum*. This model uses a texton-based library obtained using filter banks in multiple orientations and resolutions to train a deep learning neural network.

The segmentation ground truth was generated by Voronoi tessellation around cell centers manually pointed out by experts in skin research with a background in biomedical engineering and bioinformatics (Table S1 in Supplementary Material). Cell centers are used as seeds to the Voronoi tessellation. In this method, each point of the 2D Euclidean plane is assigned to a cell, such that the distance between the point and the cell seed is less than or equal to that of any other seed.

## 3 Synthetic Images

**Fig. 2:**
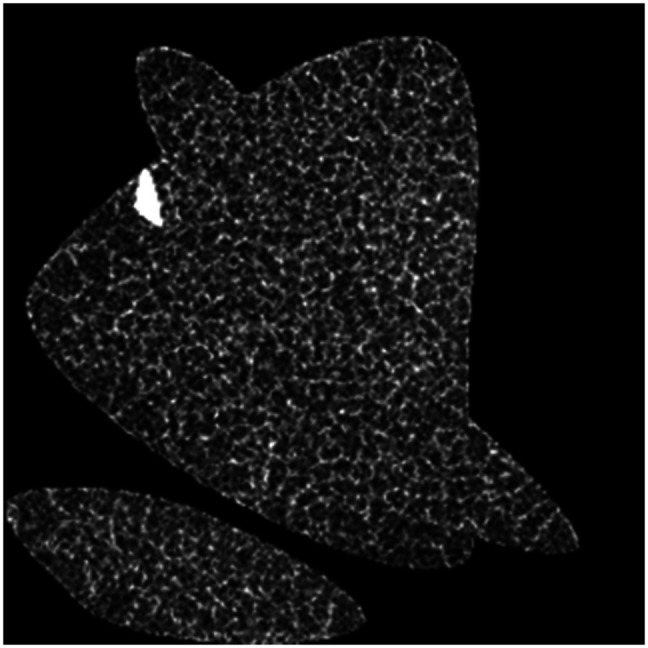
Synthetic RCM image of the *S. spinosum* created using a hard-core process, such that points are set apart with a minimum set distance from each other.

Automating cell identification in RCM images is challenging because of poor image quality due to high noise and low contrast (Table S1 in Supplementary Material). In addition, evaluating the accuracy of any method requires manual labeling to obtain a ground truth, which is subject to human error, tedious, and variable from one expert to another. To bypass these issues and guide the parameterization of our automated pipeline, we developed a process to create synthetic RCM images (see Fig. 2) that are fully user-controlled, with a perfectly annotated ground truth (*a priori* known cell centers) and not limited by the number of labeled images. These images were created by generating a random tissue mask using random Bezier curves, i.e., continuous smooth curves. Within the generated shape, seeds separated by a set distance representing cell centers were generated using a “hard core” process and used to construct Voronoi tessellations, which have been previously used to represent both skin cells^5^ and other types of cells.^9^^–^^11^ Different levels of additive Gaussian noise were then added to the created synthetic image to simulate the noise levels of a real RCM image and the heterogeneous intensity within the region of interest (ROI) by convolving the synthetic image by a heterogeneous intensity mask.

## 4 Full Image Analysis Pipeline

We present a method to automatically detect keratinocytes on confocal images based on the detection of membranes, and we compare it with two machine learning-based approaches. The first one is based on the U-Net^12^ algorithm, and the second one is based on the cell-cutter^13^ algorithm.

### 4.1 Identification of the Region of Interest

**Fig. 3:**
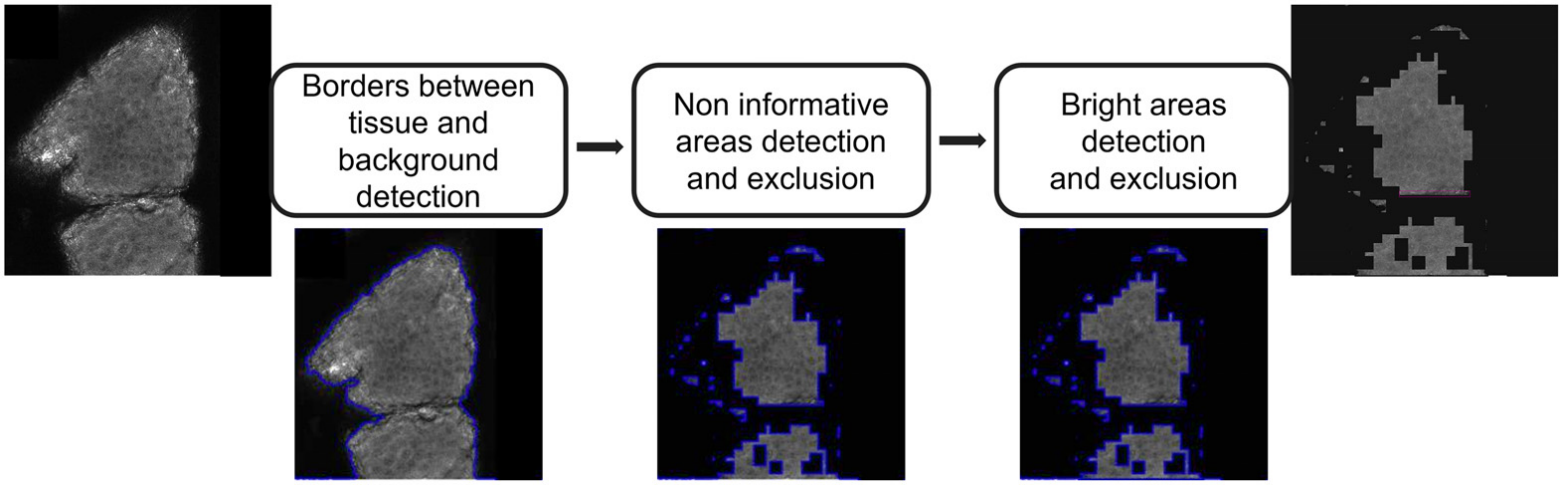
Steps of the identification of the region of interest. A morphological snake algorithm was used to identify the borders with the background, followed by a support vector machine algorithm trained to detect the noninformative areas and a succession of morphological operations to remove bright spots to an RCM image at the *S. granulosum* level. Area marked in blue, ROI mask; in pink, the area that should have been removed. Image contrast was adjusted for easier visualization.

RCM images tend to be noisy and nonuniform, which hinders the development of automated segmentation methods. To guide our cell detection, we started by identifying the ROI, i.e., the region containing epidermal cells. To do so, the black background was first identified (see Fig. 3). Islands of cells surrounded by dark empty areas are observed on RCM images, which are due to the skin microrelief lines^14^ (see Fig. 1). To identify these furrows and begin building a binary mask of the ROI, a morphological geodesic active contour^15^ algorithm (known as a snake algorithm) was applied to each image. This method employs morphological operators to detect visible contours based on their intrinsic geometric measures, even if they are noisy or partially unclear, by minimizing^16^ the energy function [Eq. (1)] assigned to a surface S, which is given as

E(S)=∬g(I)(S(a))da, (1)

where da is the Euclidean element of the area, the ROI on the image is defined by $g(I):\;R^{d}\to R^{+},x\to g(I)(x)$, and S(a) is the surface area.

After identifying the microrelief lines on each RCM image, other noninformative areas were detected. These areas are part of the tissue on RCM images and are due to low contrast and a drop in signal-to-noise ratio (see Fig. 1). A texture classification was applied to the images by training a support vector machine (see Fig. 3) on four features of the gray level co-occurrence matrix^17^ (GLCM), which successfully discriminates between the informative and noninformative areas. In the formulas below, P is the GLCM histogram used to compute each feature, for a gray level j distant from a gray level i. These features are

1. homogeneity^18^
  $$\text{homogeneity}=\overset{\text{levels}-1}{\underset{i,j=0}{\sum }}\frac{P_{i,j}}{1+{\left(i-j\right)}^{2}},$$ (2)
  which measures the closeness of the GLCM distribution to its diagonal (reflecting correlation);
2. contrast^18^
  $$\text{contrast}=\overset{\text{levels}-1}{\underset{i,j=0}{\sum }}P_{i,j}{\left(i-j\right)}^{2},$$ (3)
  which measures the local variations in the GLCM;
3. dissimilarity^18^
  $$\text{dissimilarity}=\overset{\text{levels}-1}{\underset{i,j=0}{\sum }}P_{i,j}|i-j|,$$ (4)
  which measures the similarity between pixels; and
4. energy^18^
  $$\text{energy}=\sqrt{\overset{\text{levels}-1}{\underset{i,j=0}{\sum }}P_{i,j}^{2}},$$ (5)
  which measures the signal uniformity within the area.

The third step in ROI identification was to remove the bright spots sometimes observable in RCM images (see Fig. 3). Indeed, RCM images of the *S. granulosum* and *S. spinosum* may contain bright areas due to the presence of keratin in hair shafts or from cornified cells at the periphery of the cell clusters (see Fig. 1). This was accomplished by applying a succession of dilations and erosions on the RCM image where the background and the noninformative areas were removed and which had been blurred with a Gaussian filter and binarized with a binary threshold.

### 4.2 Identification of Individual Cells

**Fig. 4:**
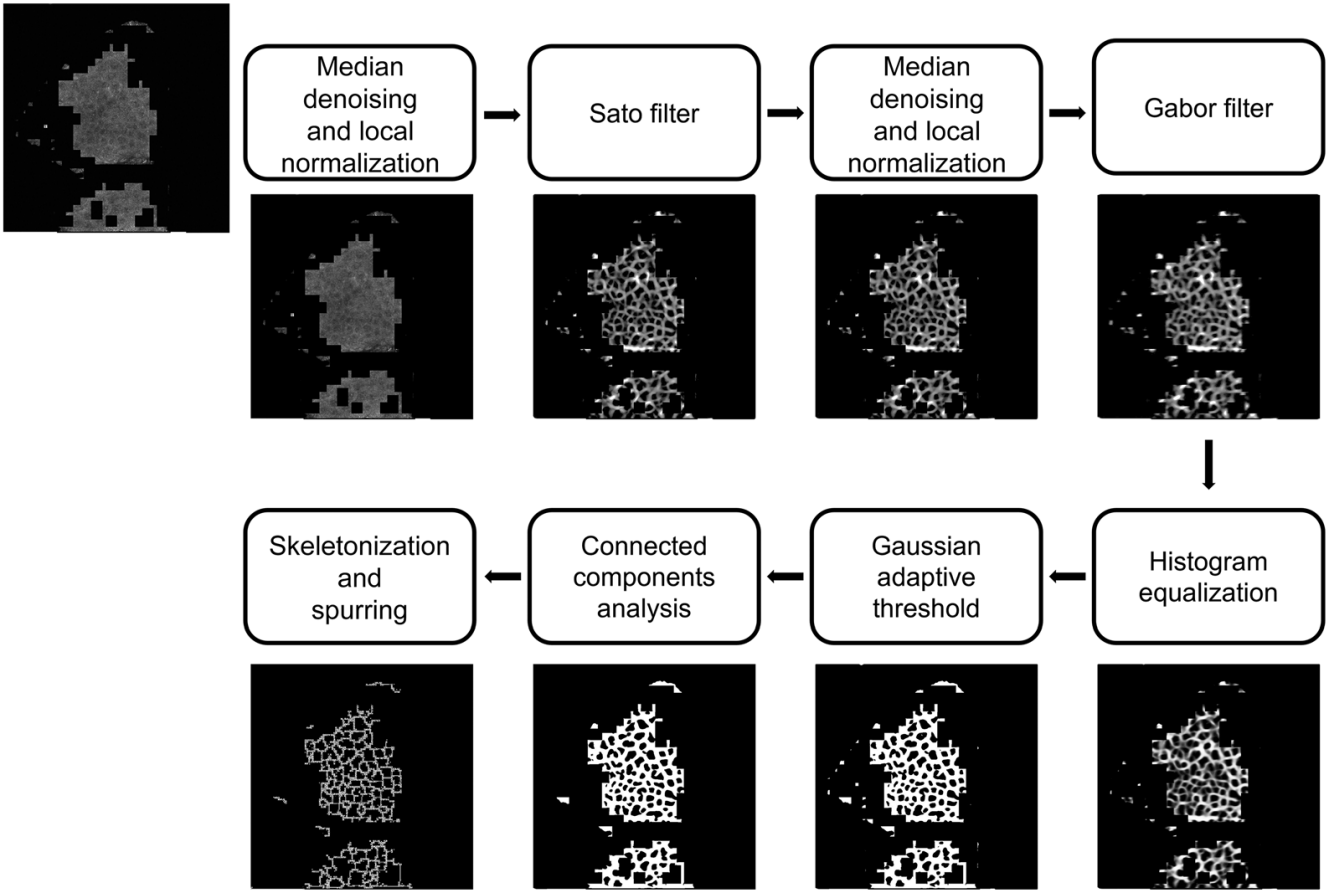
Image processing pipeline for the identification of individual cells. A median filter and a local normalization were applied to the image with the ROI mask, followed by a Sato filter. Its output was filtered with a median filter and locally normalized, and a Gabor filter was applied to it. A threshold was applied on the output after histogram equalization, and small blobs were removed with a connected components analysis. The result was then skeletonized, and spurious branches were removed. Image contrast was adjusted for easier visualization.

After identifying the ROI on the RCM image, a median filter was used to remove noise, followed by a local normalization, which renders the variance and mean of the denoised image unchanged (see Fig. 4). Then, the resulting image was filtered with the Sato tubeness filter^19^ to detect white continuous ridges, here, the bright cell membranes (see Fig. 4). The filter parameters were chosen to approximate the width and length of a cell membrane in the *S. granulosum* and *S. spinosum*. To the filter output, a median filter and local normalization were applied, while making sure that the ROI binary mask was respected (see Fig. 4). A Gabor filter was then applied to the previous image to refine membrane detection by convolving the image by a windowed signal of varying frequencies and orientations. The output of the Gabor filter was equalized with a histogram equalization to adjust the image contrast, followed by Gaussian adaptive thresholding, which dynamically and locally changes the binarization threshold over the entire image to account for changes in contrast and brightness. The local threshold value is defined as the Gaussian-weighted sum of neighboring values. This assumes that smaller regions of an RCM image are more likely to be similar. A connected-components analysis was used on the obtained binary image to remove any small blobs in the detected membranes, followed by a second connected-components analysis on the inverse of the image to close any holes in the membranes due to the graininess of the image and of the cell membranes. Finally, the clean binary image was skeletonized, and any spurious branches were removed from the skeleton. The pipeline is shown in Fig. 4.

### 4.3 Postprocessing

**Fig. 5:**
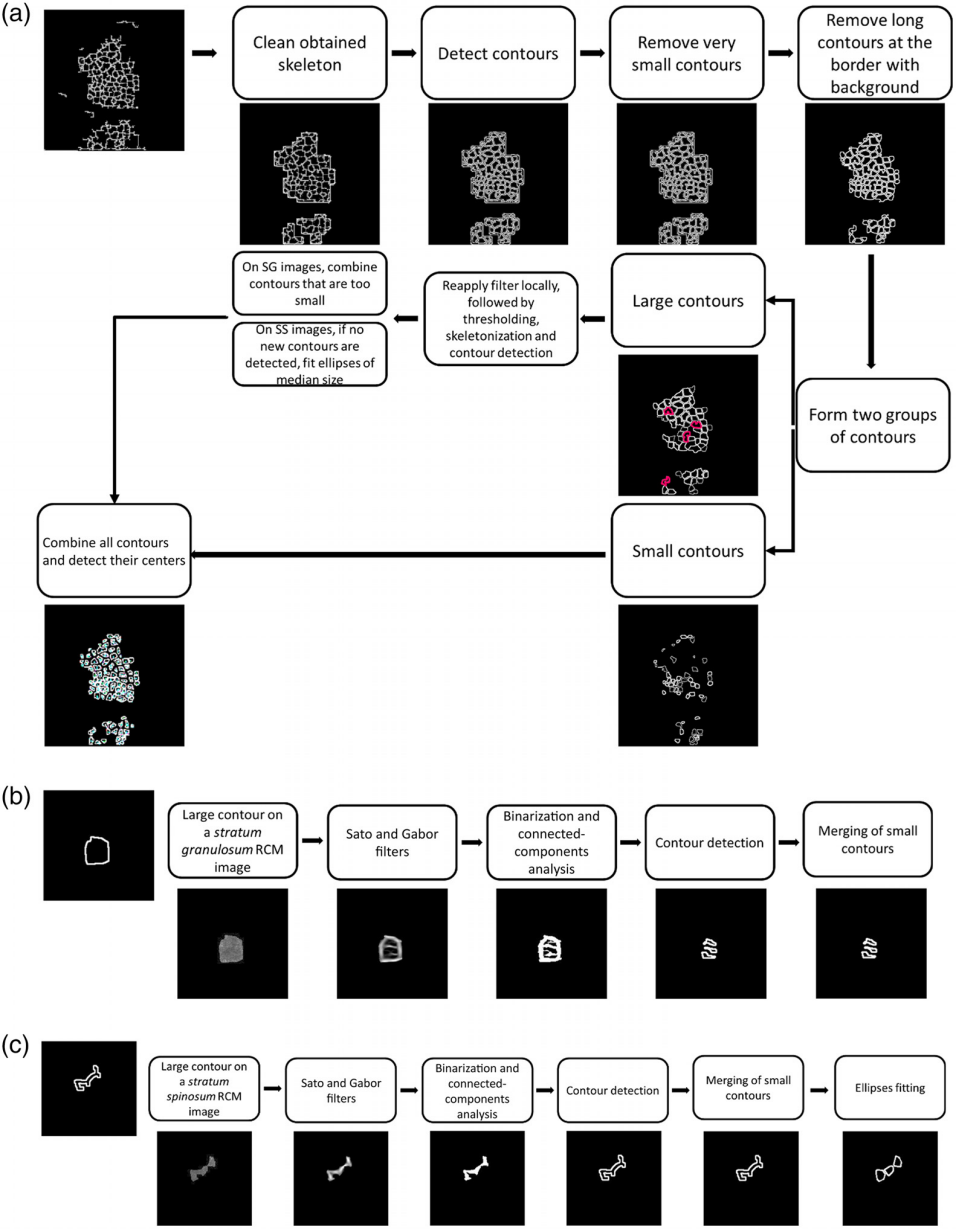
Postprocessing pipeline. (a) The skeleton obtained after the previous step was cleaned, and contours were detected. Small contours, as well as long contours close to the border with the background, were removed. The remaining contours were divided into two groups: small and big contours. Big contours were filtered again to improve the detection locally. The new resulting contours were then combined with the small contours and their centers were detected. Areas marked in pink, some contours where two cells were merged are highlighted. (b) Example of large contours improvement for a *Stratum granulosum* image. (c) Example of large contours improvement for a *Stratum spinosum* image. Image contrast was adjusted for easier visualization.

The skeleton was cleaned to remove any remaining spurs. This was accomplished by applying a morphological closing to the skeleton. Individual contours, i.e., detected keratinocytes, were detected on the skeleton. To improve the detection, very small contours were removed (area<100 for *S. granulosum* and area<50 for *S. spinosum*) as well as long contours at the border with the background, i.e., eccentricity>0.85 [see Fig. 5(a)]. These thresholds were determined empirically. The remaining contours were divided into two groups: (1) large contours with an area > 1000 for *S. granulosum* and area > 120 for *S. spinosum* and (2) small contours with an area ≤ 1000 for *S. granulosum* and area ≤ 120 for *S. spinosum*. On each area of the original image determined by a large contour, a Sato filter^19^ was applied with different parameters than previously used, i.e., smaller filter scales for more local detection of membranes. The output was then binarized with Otsu thresholding^20^ for *S. granulosum* images and with Gaussian adaptive thresholding for *S. spinosum* images. Small blobs were removed with a connected-components analysis. The subsequent binary image was skeletonized, and its contours were detected. On images of the *S. granulosum*, obtained contours with an area smaller than 110 were merged with their neighbors [see Fig. 5(b)]. On images of the *S. spinosum*, in which cells and therefore detected contours are much smaller, if the second filter iteration still failed to detect more than one contour, as many ellipses as possible were fitted within the detected contour [see Fig. 5(c)]. These new contours were then combined with the previously found small ones, and their cell centers were detected.

### 4.4 Accuracy evaluation

**Fig. 6:**
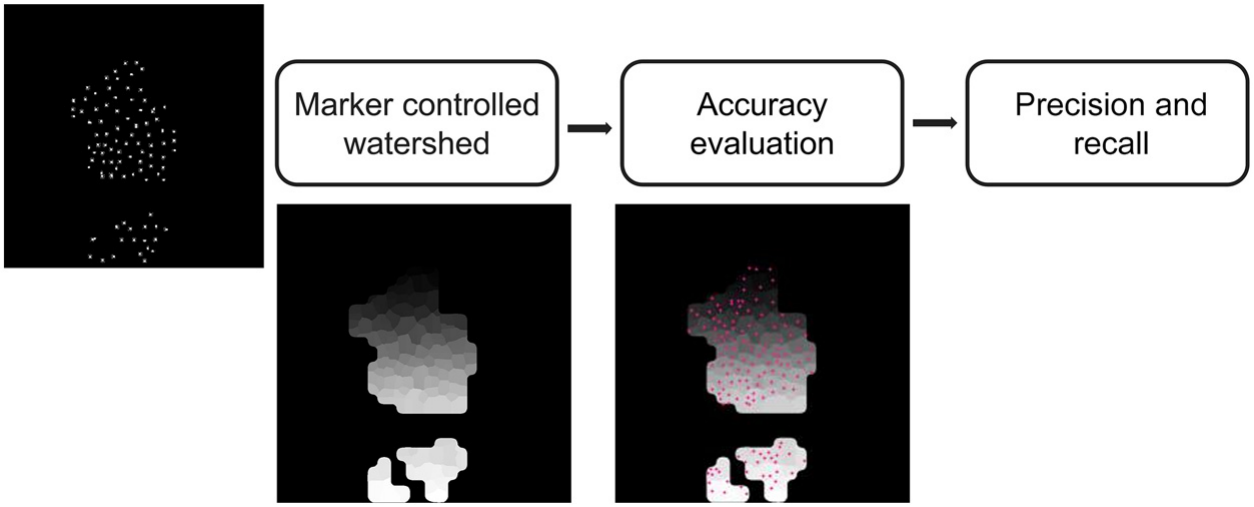
Detection accuracy evaluation pipeline. A marker-controlled watershed was applied to the detected cell centers, and the resulting labels were compared to the manually detected ground truth, which is marked in pink. The returned metrics were precision and accuracy. Image contrast was adjusted for easier visualization.

The obtained cell centers were used to initiate a marker-controlled watershed^21^ on the ROI. This method considers the input image to be a topographic surface, which is flooded starting from set seeds or markers, i.e., the detected cell centers, and returns a labeled gray-scale image, in which each label is a catching basin, i.e., a detected cell. This labeled image was then compared against manually detected cell centers using the software d-accuracy,^22^ which evaluates several indexes of the detection quality (see Fig. 6). Two accuracy metrics were evaluated: (a) precision (the fraction of correctly detected cells among all detected cells) and (b) recall (the fraction of accurately detected keratinocytes among all cells defined in the ground truth).

## 5 Results

### 5.1 On Synthetic RCM Images

To determine the minimum desired accuracy, multiple synthetic images were generated using the “hard-core” process previously described within the correct ROI mask. We placed random points using a hard-core process to mimic cell size within a correct ROI mask and generated a Voronoi tessellation around them. We then calculated the accuracies of these random detections against the absolute ground truth and obtained 60% precision and recall, which we used as the lowest accuracies threshold to better interpret our algorithm performance.

When applying the pipeline to 4 synthetic images, the median precision was 83.5% (±6.74%), and the median recall was 92.5% (±1.22%).

### 5.2 On Real RCM Images

We evaluate the performance of our approach with respect to two experts on seven real RCM images. When compared with the first expert, our cell detection approach on one image has a precision of 71.6% (±7.4%) and a recall of 84.8% (±11.9%). When compared with the second expert, our cell detection approach has a precision of 71.6% (±7.0%) and a recall of 65.9% (±15.9%) (see Table 1).

**Table 1:** Cell detection accuracy on *Stratum granulosum* and *Stratum spinosum* RCM images for two different experts. Data shown as median (±1 standard deviation).

	Precision (%)	Recall (%)
Detections versus Expert 1	71.6 (± 7.4)	84.8 (±11.9)
Detections versus Expert 2	71.6 (±7.0)	65.9 (±15.9)
Expert 1 versus Expert 2	59.4 (±8.0)	36.2 (±11.3)

When looking into the differences between the experts, we notice that Expert 2 is less sensitive in his detection, i.e., has smaller recall and, therefore, more false negatives. The obtained results are more consistent with Expert 1 and prove to be accurate compared with interexpert variability.

### 5.3 Comparison with Machine Learning-based Approaches

Although our approach gives reasonable results on RCM images of the granular and spinous layers, its performance can be hindered by the presence of cells from different epidermal layers in the same image, which makes parameterization of the different steps complicated. Our method is a multistep approach, with multiple parameters each, that each influence cell detection and its accuracy. In addition, the noise and nonuniformity of the images have a great impact on the method performance. Steps like median filtering, local normalization, and ROI determination decrease the impact of noise on the results but do not remove it completely.

The computational time (8 cores and 16 GiB of RAM) is about 10 min depending on the size of the ROI and noisiness of the image, which impacts the amount of postprocessing required, compared with 20 to 40 min for a manual annotation by an expert. Although this is a major advantage when compared with the time required to identify keratinocytes manually on RCM images, other challenges remain.

To overcome these challenges, we considered machine learning-based approaches. This shifts our paradigm from a description (meaning building knowledge or using prior knowledge of the studied structures morphological features to identify them) to a prediction (meaning training a model to discover underlying patterns in the image by minimizing differences between ground truth and prediction). By doing so, the goal was to reduce manual tuning of the approach and reduce computational time. Unfortunately, this requires a significant number of labeled images, which in our case was limited. To solve this issue, we augmented the training set with synthetic images.

We tested two machine learning-based approaches: the U-Net algorithm^12^ and the cell cutter algorithm.^13^ Images were split between training and testing with an 80:20 ratio and were the same for all U-Net models.

**Fig. 7:**
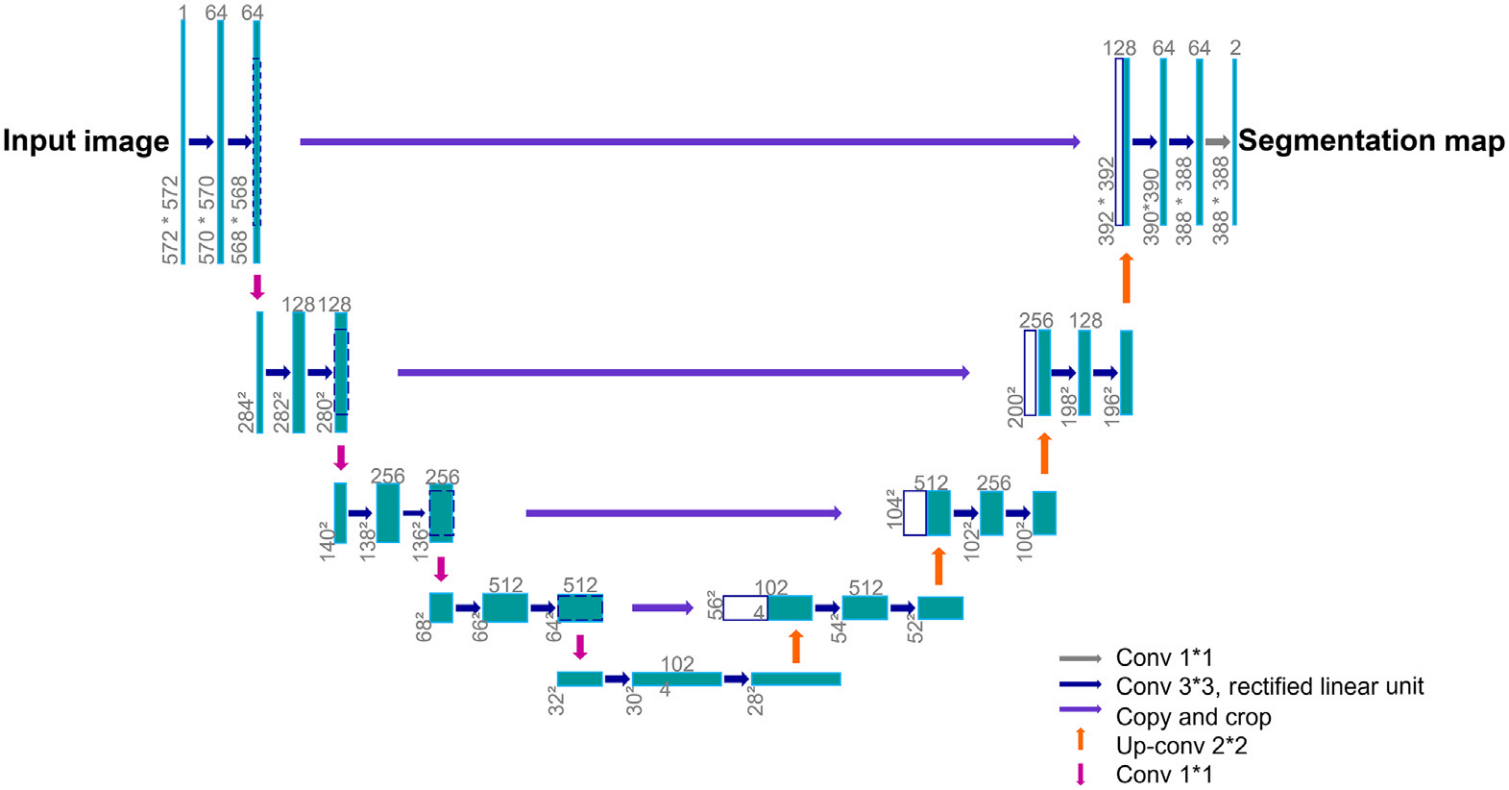
U-Net architecture. The teal box represents the feature map. The number of channels is indicated on top of the feature map box. The image size in pixels is indicated on the lower left side of the box. Boxes with dark blue contours represent copied feature maps. The arrows indicate the different operations.

U-Net^12^ is a fully convolutional neural network^23^ made of two symmetrical paths forming a U-shape (see Fig. 7). The first path is a contracting one that captures the context information and is an encoder network. It is made of a succession of 3×3 convolutions followed by a rectified linear unit and 2×2  max pooling for down sampling. Each down-sampling operation doubles the number of feature channels. The contraction reduces the spatial information while augmenting the feature information. The second path is an expanding one, i.e., a decoder network, and captures localization information. It consists of a series of up-sampling followed by a 2×2 up-convolution, which halves the number of feature channels, concatenation with the cropped feature map from the symmetrical contracting path and two 3×3 convolutions, each followed by a rectified linear unit. The large number of features in the expanding path allows the network to propagate context information through the network to higher resolution layers.

**Fig. 8:**
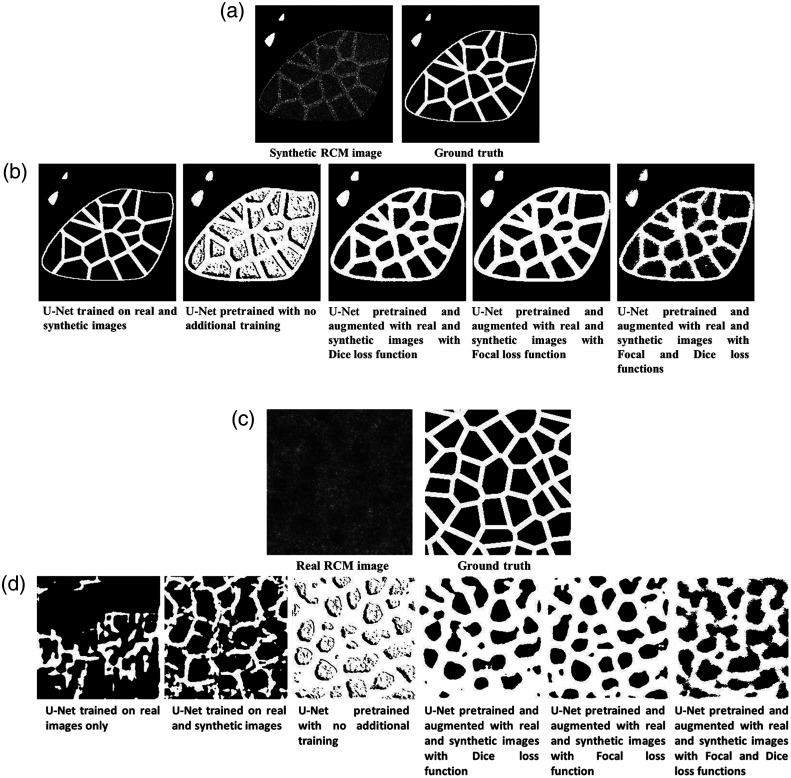
(a) Synthetic RCM images and its segmentation ground truth. (b) Tested U-Net architectures segmentations on a synthetic RCM image. (c) Real RCM images and its segmentation ground truth. (d) Tested U-Net architectures segmentations on a real RCM image.

To find the optimal U-Net configuration, we tested six models based on the same architecture and evaluated their precision, recall, and F1-score (see Table 2, Fig. 8). The first one of these models is a U-Net trained solely on 39 real RCM images (4 participants, 20 to 35 years). The limited training data are due to a lack of manually labeled data because of the time and skills required to identify keratinocytes on RCM images. This attempt proved to have a null accuracy, which was explainable by the limited training set. We therefore augmented the dataset with synthetic images. This augmentation improved both accuracy metrics on both real and synthetic images (see Table 2). However, the obtained metrics were very much unsatisfactory for real images (see Fig. 8), especially the trade-off between precision and recall. Indeed, the obtained perfect median precision and very low recall mean that very few cells are detected, but the detected ones are correct. Although this could be useful when studying individual cells, it falls short when looking at the entire tissue structure. This led us to use a pretrained U-Net model with the assumption that it would be closer to convergence and therefore would require a smaller training set, first without any additional training as an accuracy baseline and then by refining its detection with real and synthetic images and with different loss functions (see Table 1). The pretrained U-Net was trained on the 2012 ImageNet Large Scale Visual Recognition Challenge dataset^24^ with an efficientnetb3 backbone^25^ and tested with loss functions taking into account the class imbalance in RCM images, both real and synthetic, i.e., there is more background than there are cell membranes. We tested the dice loss function,^26^ which is given as

ce Loss (y,p^)=1−2yp^+1y+p^+1, (6)

where (y,p^)=(real value,predicted value),

**Table 2:** Accuracy metrics for tested U-Net models.

Model	Image dataset	Synthetic RCM images			Real RCM images		
		Median testing precision	Median testing recall	Median testing F1-score	Median testing precision	Median testing recall	Median testing F1-score
U-Net trained on real images only	Training: 39 images Testing: 10 images	0	0	0	—	—	—
U-Net trained on real and synthetic images	Training: 280 images Testing: 70 images (6 of which are real RCM images)	0.923 ± 0.092	0.958 ± 0.093	0.930 ± 0.086	1.000	0.083 ± 0.108	0.154 ± 0.156
U-Net pretrained with no additional training	Pretraining on 2012 ILSVRC ImageNet dataset, with efficientnetb3 backbone	0.027 ± 0.020	0.091 ± 0.107	0.041 ± 0,031	0.017 ± 0.008	0.125 ± 0.067	0.031 ± 0.014
U-Net pretrained and augmented with real and synthetic images with dice loss function	Pretraining on 2012 ILSVRC ImageNet dataset, with efficientnetb3 backbone Training: 203 synthetic images, 43 real images Validation: 68 synthetic images, 13 real imagesTesting: 30 synthetic images, 5 real images	0.909 ± 0.167	0.913 ± 0,107	0.911 ± 0,147	0.520 ± 0,109	0.482 ± 0.234	0.516 ± 0.164
U-Net pretrained and augmented with real and synthetic images with focal loss function		0.917 ± 0.154	0.923 ± 0.107	0.923 ± 0.136	0.500 ± 0.044	0.609 ± 0.238	0.550 ± 0.120
U-Net pretrained and augmented with real and synthetic images with focal and dice loss functions		0.909 ± 0.174	0.917 ± 0.100	0.909 ± 0.148	0.545 ± 0.159	0.571 ± 0.186	0.603 ± 0.154
FIAP	—	0.835 ± 0.067	0.925 ± 0.012	0.878 ± 0.021	0.720 ± 0.068	0.850 ± 0.110	0.779 ± 0.084

and the focal loss function^27^

$$\text{Focal Loss }(p_{t})=-\alpha_{t}{\left(1-p_{t}\right)}^{\gamma }\;\log(p_{t}),$$ (7)

where the estimated probability of class $p_{t}$ is defined as

$$p_{t}=\{\begin{array}{ll} p, & \text{if }y=1\\ 1-p, & \text{otherwise} \end{array},$$ (8)

where (y,p)=(real,prediction) and $\alpha_{t}$ and γ are weight parameters.

We finally tested a combination of the two loss functions. We obtained very close accuracy metrics with the three configurations on the synthetic images, and the best cell identification accuracies on real images using a pretrained U-Net model, augmented with real and synthetic RCM images, with the combined dice and focal loss functions (see Table 2). However, the accuracy of any of the tested U-Net approaches is lower than that of the full image analysis pipeline (FIAP) previously presented in this work, especially for real RCM images.

The second tested machine learning approach was the cell cutter^13^ algorithm, an unsupervised marker-controlled segmentation algorithm that does not require manually annotated data for training. Marker locations are generated using a real or synthetic nuclei image, and U-Net algorithms are then locally applied to each patch surrounding a marker to model cell features and produce a more accurate membrane segmentation. This localized patch approach turns a multicell segmentation problem into a multi-single-cell segmentation problem, i.e., if the marker is well defined, each patch will contain one cell and we will be looking for a single cell per patch instead of multiple cells at once; it is based on the assumption that nuclei are morphologically simpler and thus easier to accurately detect, and it aims to reduce the undersegmentation bias common to images with crowded adjacent cell populations,^28^ as long as nuclei are correctly detected. Because we do not have nuclei images matching our RCM images, we built synthetic marker images by applying the first two steps of our FIAP, i.e., ROI and individual cells identification, thus using cell cutter as a replacement for our postprocessing step. Combining these two methods into a hybrid approach aimed to be a trade-off between prediction and recall. Unfortunately, applying the cell cutter algorithm to our real RCM images failed to give satisfactory results, with the best obtained (precision, recall) = (71%, 56%), making the recall lower than the minimum accepted thresholds, i.e., the algorithm merges markers and results in lower accuracy metrics than using the FIAP previously described.

## 6 Discussion

RCM provides information on the geometrical and topological properties of the skin and how they change due to age or responding to certain stimuli, with near histological resolution. However, the study of RCM images is currently mainly done manually and therefore is tedious, time consuming, and subject to human interpretation and interexpert variability. An automated approach to extract quantitative descriptors from confocal images would enable an easier, more reproducible, precise, and rigorous study of these images and may provide metrics of interest in disease diagnosis.^7^^,^^29^

We have shown that the automated detection of keratinocytes on RCM images of the *S. granulosum* and *S. spinosum* is achievable using a method based on the morphological features of the cells, which is an important step toward the quantitative study of these images and of skin. This could help streamline RCM images analysis, thus helping to unlock actionable insights faster, both for commercial and research purposes. Our method’s results, accuracy, and computational time can be influenced by its manual parameterization, as well as the image noise and nonuniformity. To bypass these issues, we used machine learning-based approaches; however, these showed lower accuracy (see Table 3). This could be explained by the small training set of real images (39 to 43 depending on the tested model, see Table 2) and the differences with the synthetic images used to augment it. These low accuracies could also be due to using the ground truth as defined on real RCM images. This ground truth was created using Voronoi tessellation using the manually detected cell centers as seeds and thus may not perfectly match the actual membrane positions. The reasoning behind using artificially created membrane ground truth from real manually annotated cell centers was to reduce the class imbalance problem in our images, i.e., more background than cell centers and cell membranes, and thus shifting our problem from a cell center detection problem to a membrane segmentation one. Subjectivity in the manual segmentation used as ground truth may also impact accuracy metrics values, as observed in the differences between the two experts who have a similar level of training and experience with RCM images of about a decade (see Table 1). These reasons may also explain the differences in accuracies between real and synthetic RCM images (see Table 2).

**Table 3:** Comparison between the proposed methods.

	Advantages	Limitations
Manual cell identification	• Fully explainable	• Time consuming
		• Subjective
Full image analysis pipeline	• Explainable: based on knowledge of the morphological properties of the studied structures.	• Presence of multiple layers.
	• Good accuracy against manual segmentation by expert graders.	• Manual parameterization
	• Satisfactory trade-off between recall and precision.	• Image noise and heterogeneity.
U-Net	• Based on prediction and discovering patterns in the image.	• Image noise and heterogeneity.
	• Shorter computational time (excluding training time).	• Large training set size required for good results, leading to poor accuracy on real RCM images.
	• Poor trade-off between precision and recall.	
Cell cutter	• Based on prediction and discovering patterns in the image.	• Image noise and heterogeneity.
	• Shorter computational time (excluding training time).	• Large training set size required for good results, leading to poor accuracy on real RCM images.
	• Multi-single cell segmentation instead of multicell segmentation.	• Prior knowledge required: marker locations.

A prospective solution to the limited labeled data and thus the low accuracy of machine learning-based approaches could be using semisupervised learning, multitask learning, or a combination of the two. Semisupervised learning uses both labeled and unlabeled data,^30^ introducing the information from the latter into the model to improve its accuracy. Multitask learning, on the other hand, performs multiple related tasks in parallel with limited labeled data. Because the tasks are related to each other, e.g., task 1 is cell centers and task 2 is cell membranes detection, they can improve each other’s performance by constraining each other’s solution space and thus improving overall accuracy. These methods aim to improve accuracy without the use of synthetic images and, with benefiting from the information in unlabeled data in a cost-effective manner, not requiring additional manual labeling.

The proposed FIAP is now limited to the analysis of confocal images of the *S. granulosum* and *S. spinosum*. It would be biologically interesting to study images of the basal layer where cell replication occurs and several skin diseases emerge. Unfortunately, this would be challenging using RCM, whether done manually or automatically. Indeed, it would be complicated for an expert to establish a ground truth on images of the *S. basale* because of poor image quality and severe drop in the signal-to-noise ratio. Furthermore, the *S. basale* is an undulated monolayer that is not visible in a single transversal optical slice but is a ring of cells around the dermal papilla structure.

To our knowledge, only one paper has been published on the automated detection of keratinocytes in *in vivo* RCM images on the site of a melanocytic nevus, and it was based on a rotationally symmetric error function reflectance profile modeling keratinocyte shape, with fixed parameters for both *S. granulosum* and *S. spinosum* cells.^31^ The method was statistically validated, basing its accuracy on obtained cell density, whereas the proposed FIAP was validated against a manually obtained ground truth. This, we believe, makes the proposed method more accurate.

This approach based on keratinocytes morphological features will be useful in uncovering new insights in the study of skin physiology, infant skin maturation, and adult skin aging,^5^^,^^32^^–^^35^ as well as skin diseases observable with RCM, e.g., melanomas.^31^^,^^36^^–^^41^ Despite its limitations, the approach gave satisfactory results in the detection of keratinocytes on RCM images of the *S. granulosum* and *S. spinosum*, and the normalization steps helped achieve a robust parameterization of the approach for each epidermal layer. Classical machine learning approaches failed to give satisfactory results, but more advanced deep learning methods could give more accurate results in keratinocytes detection on RCM images of the *S. granulosum* and *S. spinosum*.

## Code, Data, and Materials Availability

The code, data, and materials presented in this paper are not publicly available. Requests for the code and data will be considered if received by the authors.
