# Supplementary material for: Automatic granular and spinous epidermal cell identification and analysis on in vivo reflectance confocal microscopy images using cell morphological features
Source: J Biomed Opt. 2023 Apr 8;28(4):046003. doi: 10.1117/1.JBO.28.4.046003 (PMC10082446; doi:10.1117/1.JBO.28.4.046003)
Supplement: Supplementary file 1 [file JBO_028_046003_SD001.pdf]

Supplementary material

Table S1 Summary of used and labelled data for accuracy evaluation.

**Number of manually labelled images by Expert 1**	**Number of manually labelled images by Expert 2**	**Number of generated synthetic images**
7 (all in common with Expert 2)	7	300
